# Evaluation of Effective Connectivity Between Brain Areas Activated During Simulated Driving Using Dynamic Causal Modeling

**DOI:** 10.3389/fnbeh.2020.00158

**Published:** 2020-09-23

**Authors:** Mi-Hyun Choi, Hyung-Sik Kim, Soon-Cheol Chung

**Affiliations:** Biomedical Engineering, Research Institute of Biomedical Engineering, School of ICT Convergence Engineering, College of Science and Technology, Konkuk University, Chungju, South Korea

**Keywords:** effective connectivity, driving, visual attention pathway, inhibitory control movement pathway, episodic memory retrieval pathway

## Abstract

This study was examined the effective connectivity between brain areas activated during driving. Using a driving simulator, the subjects controlled a wheel with both of their hands as well as an accelerator and brake pedal with their right foot. Of the areas activated during driving, three areas from each hemisphere were analyzed for effective connectivity using dynamic causal modeling. In the right hemisphere, bidirectional connectivity was prominent between the inferior temporal gyrus, precuneus, and lingual gyrus, which provided driving input (driving input refers to the area of input among areas connected with effective connectivity). In the left hemisphere, the superior temporal gyrus provided driving input, and bidirectional connectivity was prominent between the superior temporal gyrus, inferior parietal lobule, and inferior frontal gyrus. The visual attention pathway was activated in the right hemisphere, whereas the inhibitory control movement and task-switching pathways, which are responsible for synesthesia, were activated in the left hemisphere. In both of the hemispheres, the visual attention, inhibitory control movement, and episodic memory retrieval pathways were prominent. The activation of these pathways indicates that driving requires multi-domain executive function in addition to vision. Moreover, pathway activation is influenced by the driving experience and familiarity of the driver. This study elucidated the overall effective connectivity between brain areas related to driving.

## Introduction

The development of functional magnetic resonance imaging (fMRI) has enabled research on the function and connectivity of brain areas. Previous fMRI studies on driving, which requires complex cognitive processing, such as attention, learning, memory, and decision making, were conducted using driving simulators. Michon (1984) reported that driving requires complex cognitive processing of three interacting hierarchical levels, including the strategic (i.e., trip planning and route finding), tactical (i.e., planning of relevant actions based on the current driving context), and operational (i.e., action execution and perception) levels. Drivers should drive appropriately, paying attention to not making mistakes, which requires complex cognitive processing. Most driving accidents are caused by drivers’ mistakes in cognition and judgment, demonstrating that cognition and judgment are crucial for driving. More than 90% of the information required for such cognition and judgment during driving is acquired through vision.

In particular, many studies on changes in brain activation related to visual cognition and spatial attention during driving have been conducted (Arrington et al., 2000; Friston and Buchel, 2000; Tomasi et al., 2004). The main areas related to visual cognition are the primary visual (V1) and motion-sensitive visual regions [V5/area middle temporal (MT)] and the parietal cortex (Brodmann area 7; Friston and Buchel, 2000). Further, the brain areas related to high-order visual processing are the posterior cingulate, cerebellum, and occipital and parietal cortices (Calhoun et al., 2002). Areas related to visual attention are the occipital, inferotemporal, and parahippocampal cortices, thalamus, cerebellum, and frontal cortex (Arrington et al., 2000; Tomasi et al., 2004) and those related to spatial attention (vigilance) are the frontal and parietal cortical regions (Graydon et al., 2004). When a video game of cars was used for subjects to recognize whether the speed was slow or fast, areas related to the high-order visual, such as the occipital fusiform, cerebellum, middle and superior occipital lobes, inferior temporal lobe, and superior parietal lobe, were activated, and those related to vigilance, such as the medial, inferior, middle, superior frontal lobes, and precuneus (parietal), were activated (Calhoun et al., 2002).

Recently, there have been many studies on extraction of interaction between activated brain regions using “effective connectivity” for various cognitive performances and on direction and connection strength between regions. Studies on effective connectivity for cognitive processing are also being conducted, but there are not many studies on effective connectivity between areas that are activated during driving. In particular, Wang et al. (2015) conducted a driving experiment with drivers and non-drivers and reported greater functional connectivity in the left fronto-parietal and primary visual resting-state networks (RSNs) in people with more driving experience. The left fronto-parietal network is a connectivity related to higher-order cognitive functions, and the primary visual resting-state networks is a network related to functions of visual cognition. The driving behavior altered the functional connectivity between the cognitive and sensory intrinsic connectivity networks (ICNs), and the strength of specific connections between the left fronto-parietal and primary visual network significantly correlated with the number of years as a taxi driver (Wang et al., 2015). Shen et al. (2016) reported that the strength of connectivity between areas in the vigilance network decreased with increasing driving experience. The vigilance network is a network containing areas of anterior cingulate cortex and anterior insula. The vigilance is the ability to sustain attention over prolonged periods of time. Among the cognitive types that may appear when driving, only the results of studies on the above-mentioned networks have been reported using functional connectivity analysis.

The aforementioned studies investigated differences in functional connectivity between brain areas during driving in certain subject groups and for certain cognitive aspects, and research on overall brain connectivity during driving has so far been lacking. Particularly, connectivity among the left, right, and bilateral hemispheres during driving, their meaning and input areas, and directivity and correlation between input and other areas are yet to be investigated; however, such information can be obtained through an effective connectivity analysis.

Based on other studies and previous studies from our research team, we expect the following results on brain effective connectivity when driving. As mentioned above, since driving requires complex cognitive processing, such as attention, learning, memory, and decision making, we expect that certain cognitive areas would appear dominant in the left and right hemispheres when driving. In the right hemisphere, connectivity between areas related to the high-order visual and concentration would be dominant, and in the left hemisphere, connectivity between areas related to synesthesia and motion control is expected to be large. In addition, because the steering wheel is controlled with both hands, the motor cortex areas of the left and right hemispheres would be activated simultaneously, and since the right foot is used to operate the pedal, the motor cortex of the parietal lobe in the left hemisphere would be predominantly activated.

To investigate the correlation between brain areas activated during driving, this fMRI study analyzed effective connectivity between areas in the left, right, and bilateral hemispheres using dynamic causal modeling (DCM).

## Materials and Methods

### Subjects

Fifteen adult men (mean age: 26.0 ± 1.4 years old), without any history of mental or neurological disease and with a mean driving experience of 2.5 ± 1.6 years, were selected as subjects. All subjects were right-handed as a result of the revised Edinburgh Reading Test (Oldfield, 1971). Individuals with metal inside their bodies (e.g., cardiac pacemaker or medical wiring), which could interfere with magnetic resonance (MR) imaging, as well as those with claustrophobia were excluded. External factors, such as smoking, alcohol consumption, and coffee intake, which can influence driving and brain activation, were restricted in the subjects prior to the experiment. The purpose and details of the experiment were explained to the subjects. Practice driving was conducted until the subjects became familiar with the driving simulator environment and could drive without any accidents.

### MR-Compatible Driving Simulator

**FIGURE 1:**
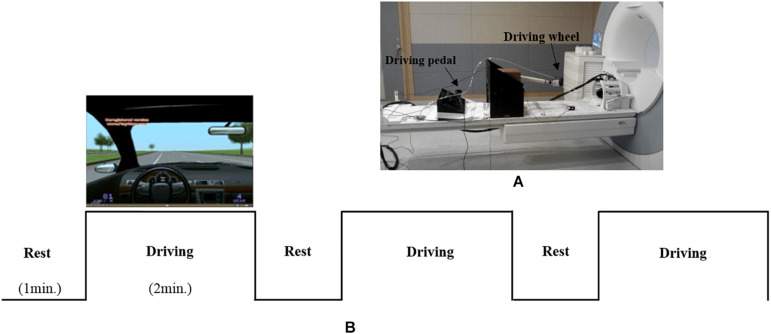
**(A)** an MR-compatible driving simulator consisting of a wheel and pedals (accelerator and brake). **(B)** The driving environment and experimental design.

As shown in Figure 1A, an MR-compatible driving simulator consisting of a wheel and pedals (i.e., accelerator and brake) was used for this study (Kim et al., 2020). The driving environment (Figure 1B), which mostly consisted of straight streets without many visual distractors, was presented using Lightrock Entertainment software. The subjects controlled the wheel with both of their hands as well as the accelerator and brake with their right foot. The subjects were asked to drive at a constant speed of 80 km/h without changing lanes. Visual information for driving was presented to the subjects through the visual system attached to the head coil. Visual system is 800 × 600 pixels, aspect ratio is 4:3, and FOV is 30° horizontal/23° vertical.

### Experimental Design

As shown in Figure 1B, the experiment consisted of three blocks, with each block consisting of rest (1 min) and driving (2 min) phases. During the rest phase, the subjects were asked to look at a fixed screen without driving. During rest phase, subjects placed both hands on the steering wheel and a right foot on the pedal without any movement. During the driving phase, the subjects were asked to drive at a constant speed of 80 km/h. To help the subjects maintain a speed of 80 km/h, speed information was presented on the lower left corner of the simulator screen. During the driving phase, alerts signaling the start (i.e., “please start driving”) and completion (i.e., “please stop driving”) of the driving task were orally provided by a researcher through the headset worn by each subject. Oral driving cues were given to subjects at each driving phase.

### Image Acquisition

Images were acquired with a 3T MRI scanner (Magnetom TrioTim, Siemens Medical Systems, Erlangen, Germany) using a standard 32-channel head coil. Single-shot echo planar fMRI scans were acquired in 29 continuous slices, parallel to the anterior commissure-posterior commissure line. The fMRI parameters were as follows: TR/TE = 3000/30 ms, FOV = 200 mm, flip angle = 90°, matrix = 128 × 128, slice thickness = 5 mm, and voxel size = 1.6 × 1.6 × 5.0 mm. Anatomical images were obtained using a T1-weighted 3D-MPRAGE sequence with TR/TE = 1900/2.48 ms, FOV = 200 mm, flip angle = 9°, matrix = 256 × 256, slice thickness = 1 mm, and voxel size = 0.8 × 0.8 × 1.0 mm.

### Image Analysis

The fMRI data were analyzed with Statistical Parametric Mapping (SPM) 8 software (Wellcome Department of Cognitive Neurology, London, United Kingdom). All functional images were aligned with anatomic images using affine transformation routines built into SPM 8. The realigned scans were co-registered to anatomic images obtained within each session and normalized to a template image in Montreal Neurologic Institute (MNI) space. Motion correction was done using a Sinc interpolation. Time-series data were filtered with a 240 s high-pass filter to remove artifacts due to cardiorespiratory and other cyclical influences. The functional map was smoothed with an 8 mm isotropic Gaussian kernel prior to statistical analysis. Statistical analysis was performed at the group level using the general linear model and theory of Gaussian random fields implemented in SPM8. A group analysis was performed to extend the inference of individual activation to the general population from which the subjects were drawn. This will list all clusters above the chosen level of significance as well as separate (>8 mm apart) maxima within a cluster, with details of significance thresholds (height threshold *T* = 4.69 (*p* < 0.05), extent threshold *k* = 0 voxels) and search volume underneath.

Subtraction method was used to obtain the activated area in the driving phase compared to the rest phase (Driving phase – Rest phase). This result is a functional map obtained through group analysis. It may be that, due to this extraction method, driving-like response from the previous driving phase was minimized in the rest phase.

### Connectivity Analysis

To extract the effective connectivity between brain areas activated during driving, DCM was used to investigate the correlation between areas of interest. DCM, which is a model-based analysis method, can be applied not only to the analysis of brain activation through general linear modeling (GLM), but also to the analysis of brain area connectivity. In this analysis, the relationship between each variable is estimated through covariate or linear regression analysis, and a model of the correlation between brain areas is constructed based on this information. For DCM analysis, models are defined in SPM8 based on MATLAB, which is followed by variable estimation and Bayesian model selection (BMS). Operating under the hypothesis that all activated areas form a network, DCM analyzes the correlation between areas with blood oxygen level-dependent (BOLD) signals and establishes optimal dynamic causality models (Friston et al., 2003).

**FIGURE 2:**
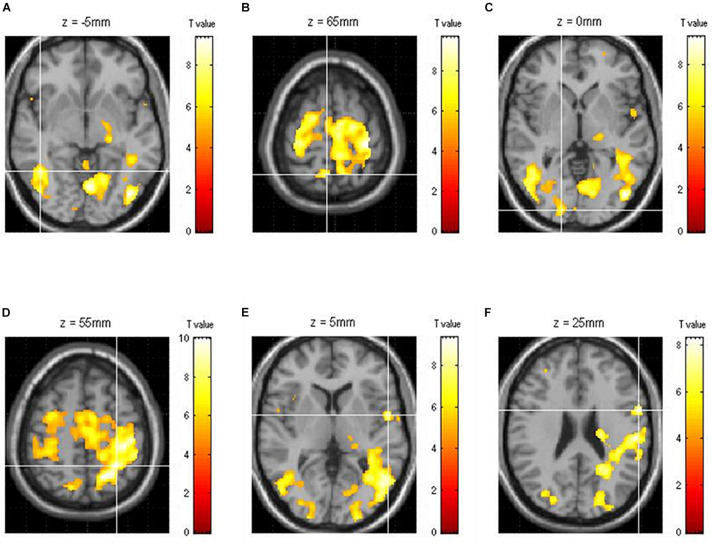
Functional brain map showing the average of all subjects obtained through group analysis. The three right [**(A)** ITG, inferior temporal gyrus; **(B)** PCu, precuneus; **(C)** LiG, lingual gyrus] and left [**(D)** IPL, inferior parietal lobule; **(E)** STG, superior temporal gyrus; **(F)** IFG, inferior frontal gyrus] hemispheric areas with the highest *z*-scores during driving.

Of the areas activated during driving, three areas from each hemisphere with the highest *z*-scores had their effective connectivity analyzed. As discussed in the results section, the three areas from the right hemisphere with the highest *z*-scores were the inferior temporal gyrus (ITG), precuneus (PCu), and lingual gyrus (LiG), whereas those from the left hemisphere with the highest *z*-scores were the inferior parietal lobule (IPL), superior temporal gyrus (STG), and inferior frontal gyrus (IFG; Figure 2). The effective connectivity was analyzed for the three areas in the left and right hemispheres as well as for all six areas in both hemispheres. Effective connectivity analysis involved the selection of driving input areas as areas of interest and modeling connectivity based on the correlation between the BOLD signals of the areas of interest. For connectivity analysis, the time-series of the BOLD signal of each area of interest was extracted from 5 mm diameter spherical regions centered around the voxel with the greatest *z*-score.

Specifically, effective connectivity analysis began by selecting driving inputs to the left and right hemispheres (i.e., three areas from each) as well as both hemispheres (i.e., six areas). After selecting areas of interest as fully connected (i.e., full bidirectional connection between all areas of interest), models hypothesizing each area as the input were established. For example, the inferior temporal gyrus, precuneus, and lingual gyrus of the right hemisphere were selected as fully connected, and three models, in which each area was set as the driving input, were established. Subsequently, using BMS, the most significant driving input model was selected using fixed effect calculations.

**FIGURE 3:**
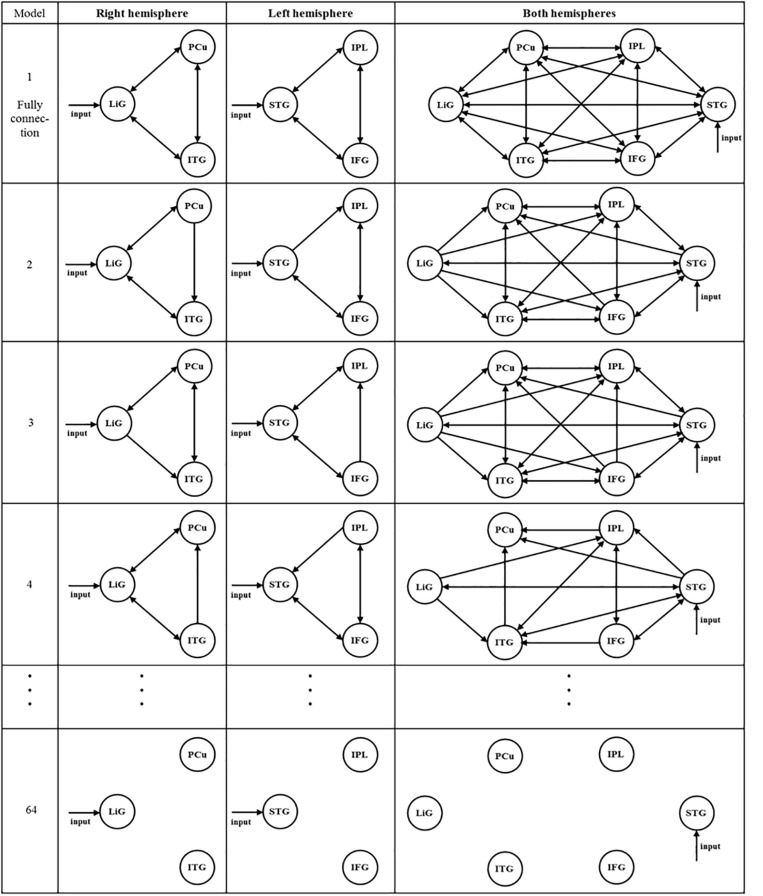
After selecting the driving input areas of the right, left, and both hemispheres, 64 models were established to analyze the connectivity between the areas of interest.

After selecting the driving input areas of the right, left, and both hemispheres, the connectivity between areas of interest was analyzed. Sixty-four models for each hemisphere were established to investigate the connectivity between the three areas of the left and right hemispheres (Figure 3). In Figure 3, the first and second columns are models of the three areas of the right and left hemispheres, respectively, and the third column shows models of the six areas of both hemispheres. As shown in the first and second columns of Figure 3, Model 1 is a full connection model indicating intrinsic connection with bidirectional connections between all areas. Moreover, Models 2–63 differ in the direction of connections while considering external connections. Model 64 has no connections between areas.

A total of 299 models of the connectivity between the six areas in both hemispheres were established (see Figure 3, third column). Similar to the models within the first and second columns, in the third column, Model 1 is a full connection model, Models 2–298 differ in the connectivity between areas of interest, and Model 299 has no connectivity.

This analysis was performed for each subject. The posterior model probability for each model was extracted for each subject using BMS fixed effects (FFX) to compare models in each hemisphere. Based on the data from each subject, group comparison of models was performed using BMS random fixed effects (RFX). RFX analysis obtains the optimal probability for presumed models, and was used to estimate the probability of each model. Model probability was tested at the group level, and the model with the highest probability was used to derive the mean correlation between areas and determine the effective connectivity.

## Results

**FIGURE 4:**
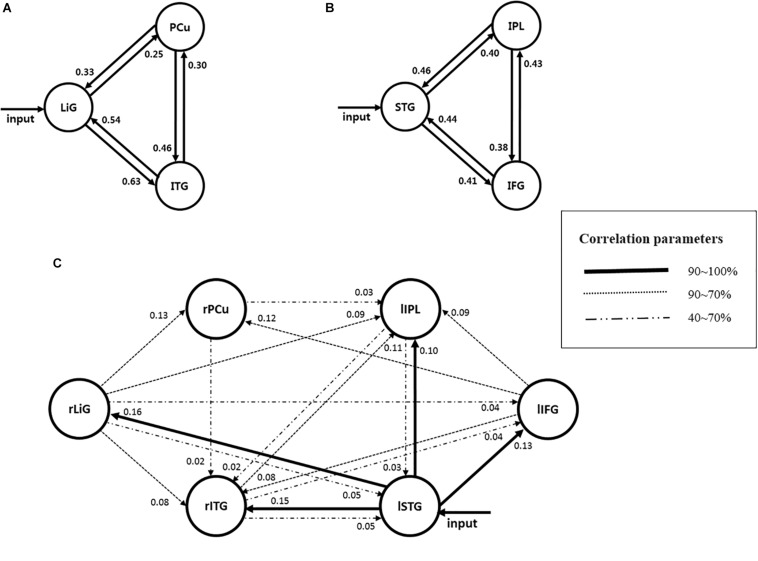
Models estimating the effective connectivity between areas activated during driving in the **(A)** right, **(B)** left, and **(C)** both hemispheres.

Of the brain areas activated during driving, the three areas in the right hemisphere with the highest *z*-scores were the ITG, PCu, and LiG (Figures 2A–C), which had *z*-scores of 9.33, 8.28, and 8.05, respectively. The most significant of the three models, in which each area was set as the driving input, was that with the LiG as the driving input (probabilities: 1.00, C-direct effects: 0.1 Hz). The connectivity between these three areas was bidirectional and had significant effects (Table 1 and Figure 4A).

**TABLE 1:** Correlation between three left (IPL, inferior parietal lobule; STG, superior temporal gyrus; IFG, inferior frontal gyrus; and right (ITG, inferior temporal gyrus; PCu, precuneus; LiG, lingual gyrus) hemispheric areas activated during driving.

	From		
	ITG	PCu	LiG
**Right hemisphere**			
*To*			
ITG		0.46 (97%)	0.63 (100%)
PCu	0.3 (93%)		0.25 (90%)
LiG	0.54 (100%)	0.33 (91%)	

	**From**		
	**IPL**	**STG**	**IFG**

**Left Hemisphere**			
*To*			
IPL		0.4 (99%)	0.43 (97%)
STG	0.46 (98%)		0.44 (98%)
IFG	0.38 (96%)	0.41 (98%)	

*ITG, Inferior Temporal Gyrus; PCu, Precuneus; LiG, Lingual Gyrus; IPL, Inferior Parietal Lobule; STG, Superior Temporal Gyrus; IFG, Inferior Frontal Gyrus.*

The three areas in the left hemisphere with the highest *z*-scores were the IPL, STG, and IFG (Figures 2D–F), which had *z*-scores of 9.91, 7.14, and 7.66, respectively. The most significant of the three models analyzed was that with the STG as the driving input (probabilities: 1.00, C-direct effects: 0.13 Hz). Similar to the right hemisphere, the connectivity between these three areas was bidirectional and had high correlations (Table 1 and Figure 4B).

For both of the hemispheres, the effective connectivity was analyzed between the right ITG (rITG), right PCu (rPCu), right LiG (rLiG), left IPL (lIPL), left STG (lSTG), and left IFG (lIFG; Table 2 and Figure 4^©^). Of the six models with each of the six areas set as the driving input, the most significant model was that with the lSTG as the driving input (probabilities: 1.00, C-direct effects: 0.15 Hz). There was prominent connectivity from the lSTG to the rITG (A-intrinsic connections: 0.15, correlation parameters: 100%), rLiG (0.16, 100%), lIPL (0.1, 99%), and lIFG (0.13, 100%).

**TABLE 2:** Correlation between six left and right hemispheric areas (rITG, right inferior temporal gyrus; rPCu, right precuneus; rLiG, right lingual gyrus; lIPL, left inferior parietal lobule; lSTG, left superior temporal gyrus; lIFG, left inferior frontal gyrus) activated during driving.

	From					
	rITG	rPCu	rLiG	lIPL	lSTG	lIFG
**To**						
rITG		0.02 (56%)	0.08 (73%)	0.02 (71%)	0.15 (100%)	0.08 (71%)
rPCu	0		0.13 (87%)	0	0	0.12 (85%)
rLiG	0	0		0	0.16 (100%)	0
lIPL	0.11 (80%)	0.03 (58%)	0.09 (75%)		0.1 (100%)	0.09 (74%)
lSTG	0.05 (64%)	0	0.05 (64%)	0.03 (60%)		0
lIFG	0.04 (63%)	0	0.04 (62%)	0	0.13 (100%)	

*r, right hemisphere; l, Left hemisphere.*

## Discussion

For driving, various cognitive processes, such as vision, synesthesia, motion control, judgment, concentration, attention, and memory, are required. In previous studies, visual network (Wang et al., 2015), vigilance network (Shen et al., 2016), and left fronto-parietal network (Wang et al., 2015) among the brain networks for various cognitive types that may appear when driving is reported using functional connectivity. The main difference between this study’s results and previous studies is to be mentioned in three ways. First, by using effective connectivity, the meaning of connectivity and the input area in each connectivity are presented. Second, the results for directionality and connection strength from the input region to other regions are presented. Third, these results are reported as brain networks that predominate in each of the left and right hemispheres. This study sought to analyze the connectivity between brain areas responsible for cognitive processing during driving in the right, left, and both hemispheres.

### Effective Connectivity Between Areas Activated in the Right Hemisphere

In the right hemisphere, the LiG, which processes visual linguistic information and plays a crucial role in the analysis of encoded visual memories (Mechelli et al., 2000), PCu, which is related to recollection and memory as well as the integration of information relating to environment perception (Lundstrom et al., 2005; Cavanna and Trimble, 2006), and ITG, which is related to higher-level visual processing (Kolb and Whishaw, 2014) had significant bidirectional connectivity. Since the right hemisphere visually perceives the driving environment and processes information for this purpose, the LiG would have been selected as the input area. Previous fMRI studies have reported that the aforementioned three areas form the visual attention pathway (Milner and Goodale, 1998; Macaluso et al., 2000; Purves et al., 2008). Visual attention can best be defined as a family of processing resources or cognitive mechanisms that can modulate signals at almost every level of the visual system. Research shows that visual attention can perform this function by actively suppressing irrelevant stimuli or by selecting potentially relevant stimuli. The connectivity from the LiG to the PCu is the dorsal stream pathway, which processes the location of objects (Milner and Goodale, 1998). Moreover, this pathway has been reported to analyze motion as well as the spatial relationship (i.e., “where”) between objects, thus being responsible for visual synesthesia (Purves et al., 2008). The connectivity from the LiG to the ITG is the ventral stream pathway, which processes information on “what” an object is (Milner and Goodale, 1998). This pathway has been reported to be responsible for high-resolution vision (Purves et al., 2008). This study, which analyzed brain connectivity related to driving, also clearly observed visual attention pathways related to the “where” and “what” of an object as in previous studies. This study also found significant bidirectional connectivity between the PCu and the ITG. Although a pathway between these two areas has not been previously reported, this finding is reasonable given the functions of each area. Information processing for driving is mostly performed through vision, and visual information is processed through simple and higher-order processing. High-resolution visual processing is necessary for driving, and recollection and memory as well as the integration of information relating to the perception of the environment play important roles in driving tasks. Therefore, the PCu and ITG would have had a significant correlation. In addition to the aforementioned functions, the PCu is also associated with episodic memory retrieval (Lundstrom et al., 2005) and vigilance performance (Shen et al., 2016). Vigilance, which is a fundamental component of attention, refers to the ability to maintain attention over a long period of time. Vigilance is crucial in driving, in which an individual must continuously monitor and react to rare signals while ignoring irrelevant stimuli. Therefore, it is possible that the PCu and ITG had a significant correlation not only because drivers perceive the driving environment based on higher-order visual processing, but also because their episodic memories and vigilance influence driving. Thus, the connectivity between the LiG, PCu, and ITG during driving, with the LiG as the input area, may serve as the visual attention-(episodic) memory retrieval pathway.

### Effective Connectivity Between Areas Activated in the Left Hemisphere

In the left hemisphere, the STG, which is primarily involved in auditory recognition and understanding language meaning (Howard et al., 2000), IFG, which is associated with information selection and monitoring as well as cognitive control (Lundstrom et al., 2005; Grindrod et al., 2008), and IPL, which is associated with perspective difference cognition,^2^ spatial cognition, and visually guided movement (Andersen, 2011; Hadjidimitrakis et al., 2012, 2019; Yttri et al., 2014; Kaas and Stepniewska, 2016), had significant bidirectional connectivity. In particular, a study reported that inferior frontal junction area (IFJ) (located at the junction of the inferior frontal sulcus and the inferior precentral sulcus), which includes the IFG region, has three main component processes (task switching, inhibitory control and working memory) (Brass et al., 2005; Derrfuss et al., 2005, 2009; Levy and Wagner, 2011; Kim et al., 2012).

Since oral driving alerts (i.e., “Please start driving” and “Please stop driving”) were provided by a researcher to the subjects during each phase, the STG, which is associated with language processing, would have been selected as the input area. The connectivity between the STG and IFG has previously been reported as a wide language network (Jeong et al., 2009); however, these areas may have driving functions as well. The STG and IFG have been associated with convergent semantic processing, which controls, suppresses, and modulates various options to successfully perform multiple related tasks (Friederici et al., 2003). Since this study required the subjects to maintain their driving lane and speed, they had to simultaneously control the wheel and pedals, which required convergent semantic processing. This task led to significant bidirectional connectivity between the STG and IFG, in which these areas formed a network associated with inhibitory control in addition to language processing. Inhibitory control, also known as response inhibition, is a cognitive process and more specifically, an executive function – that permits an individual to inhibit their impulses and natural, habitual, or dominant behavioral responses to stimuli (a.k.a. prepotent responses) in order to select a more appropriate behavior that is consistent with completing their goals (Diamond, 2013; Ilieva et al., 2015). Inhibitory control revealed that frontal, subcortical, insula (INS), and parietal regions are active.

The connectivity between the STG and IPL can be predicted according to the following observations. First, the dorsal part of the left temporo-parietal junction (TPJ) is activated during perspective tasks (Goel et al., 1995; Ruby and Decety, 2003), which require tracking of potential, or actual, perspective differences (Arora et al., 2015). The driving images presented in this study were similar to actual driving environments, requiring spatial perception of near and far perspectives. Since oral driving cues (lSTG) and driving images with perspective differences (IPL) were used for this driving task, bidirectional connectivity between these two areas would have been significant. Second, the driving cues (STG) as well as the spatial cognition and hand and leg movements needed to control the wheel and pedals for visually guided driving (lIPL) are predictive of these areas having significant bidirectional connectivity.

The bidirectional connection between the Inferior Frontal Gyrus and the Inferior Parietal Lobule may be related to movement control for controlling the steering wheel and pedals when driving. By initiating and modulating cognitive control abilities, the fronto-parietal network (Sundermann and Pfleiderer, 2012) is involved in a wide variety of tasks. Thus, cognitive control of driving by the IFG as well as control of the wheel and pedals to maintain speed by the IPL led to significant connectivity between these two areas.

Due to the use of oral driving cues, the STG was selected as the input area. The overall connectivity of the STG with the IFG and IPL can be interpreted in terms of movement during driving. First, the connectivity from the STG to the IFG, and then to the IPL, selects and monitors driving information, and permits driving (IPL) through inhibitory control (IFG). Therefore, this pathway could serve as an inhibitory control movement pathway. With the STG as the input, the IPL performs driving through spatial recognition and vision. Moreover, the cognitive control of the IFG switches between different tasks, such as controlling the wheel and pedals. Consequently, this pathway could serve as a task-switching pathway. Although these pathways may be considered identical since they both regulate movements associated with driving, they still differ in terms of whether a driving motion is performed.

### Effective Connectivity Between Areas Activated in Both Hemispheres

Of the six areas activated in both hemispheres, the lSTG was selected as the input area since oral driving cues were provided to the subjects during each phase. The following pathways, with the input area as the start and correlations above 70%, are explained below (Figure 4):

① As described previously, the connectivity between the lSTG → lIFG → lIPL is the inhibitory control movement pathway. In the left hemisphere, inhibitory control movement and task-switching pathways were observed, whereas, in both hemispheres, the inhibitory control movement pathway was dominant.
② There was a prominent connectivity between the lSTG → lIFG → rPCu. Previous studies have reported that the lIFG and rPCu were activated during episodic memory retrieval (Lundstrom et al., 2005). This pathway would likely act to select and monitor information on certain driving aspects (i.e., maintenance of speed and lane) acquired prior to driving and apply the subject’s episodic memory. Therefore, this pathway could act as an episodic memory retrieval pathway.

③ The pathway between the lSTG → rITG → lIPL could have resulted from the subjects using higher-order visual functions to perceive the driving environment (Kolb and Whishaw, 2014), spatial cognition (Andersen, 2011), and perspective differences cognition for driving.
④ The pathways between the lSTG → rLiG → rPCu and ⑤ lSTG → rLiG → rITG are visual attention pathways (see section “Effective Connectivity Between Areas Activated in the Right Hemisphere”) that were dominant in the left and right hemispheres.
⑥ The pathway between the lSTG → rLiG → lIPL → rITG appeared since the perception of perspective differences and spatial cognition (lIPL) were added to the visual attention pathway (i.e., ⑤).

## Conclusion

This study investigated the effective connectivity between brain areas activated during driving for the left, right, and both hemispheres.

Since visual cognition and processing are crucial for driving, the visual attention pathway was prominent in the right and both hemispheres. Moreover, the inhibitory control movement and task-switching pathways, which are related to synesthesia required for driving, were prominent in the left hemisphere. An interesting finding of this study was the observation of the inhibitory control movement pathway, which was prominent in the left and both hemispheres. Although research on inhibitory control has been largely conducted using go/no-go tasks (Chikazoe, 2010; Ma et al., 2015), no reports associated with driving have been made. Inhibitory control is a multi-domain executive function critical for flexible responsivity to changing task demands, and, thus, is an essential component of adaptive behavioral regulation. As expected, pathways regulating movement through inhibitory control were prominent during driving.

The episodic memory retrieval pathway observed in the right and both hemispheres is associated with drivers recalling their own experiences, indicating that driving is influenced by driving experience and familiarity.

In accordance with the hypothesis proposed in this study, connectivity between areas related to specific cognition in the right and the left hemispheres was predominant, but we could also observe interesting results that were not consistent with the hypothesis. Because both hands and right foot were used, we expected that activation would be dominant in the area including the premotor cortex and supplementary motor area. However, lIPL parietal cortex, the area responsible for controlling the movement by perceiving the situation through spatial and visual perception (controlling steering wheel with both hands and pedal with right foot in this study), was predominantly activated. As such, we suggest that IIPL cortex, which is involved in complex cognitive processing that controls behavior according to the surrounding environment, is more activated than motor cortex such as premotor cortex and supplementary motor area.

Although the driving simulator was similar to actual driving conditions, it still differed from actual conditions. Moreover, events that were not directly associated with driving, such as the oral driving cues, were included in the experiment. However, this study is still significant by being the first to investigate the overall effective connectivity between brain areas associated with driving.

## Data Availability Statement

The datasets generated for this study are available on request to the corresponding author.

## Ethics Statement

The studies involving human participants were reviewed and approved by the Institutional Review Committee of Konkuk University. The patients/participants provided their written informed consent to participate in this study.

## Author Contributions

M-HC conceived the project and performed the experiments. H-SK made the experimental system. M-HC and S-CC designed the experiments and wrote the manuscript. All authors read and edited the manuscript prior to publication.

## Conflict of Interest

The authors declare that the research was conducted in the absence of any commercial or financial relationships that could be construed as a potential conflict of interest.

## Footnotes

2 With “perspective difference cognition” or, for short, “perspective tracking” we want to merely grasp the existence of this concept required for registering an actual or potential conflict between perspectives. The more common term “perspective taking” suggests the ability to put oneself into another perspective than the perspective one currently has. This would require the tracking of a particular perspective not just the tracking of a potential perspective difference. There is growing evidence that the dorsal part of the left temporo-parietal junction (TPJ), which overlaps with the left inferior parietal lobe (IPL), is reliably activated by perspective tasks (Goel et al., 1995; Ruby and Decety, 2003).

## References

[B1] Andersen R. A. (2011). Inferior parietal lobule function in spatial perception and visuomotor integration. *Comprehens. Physiol.* 11 483–518.

[B2] Arora A. Weiss B. Schurz M. Aichhorn M. Wieshofer R. C. Perner J. (2015). Left inferior-parietal lobe activity in perspective tasks: identity statements. *Front. Hum. Neurosci.* 9:360. 10.3389/fnhum.2015.00360 26175677 PMC4485079

[B3] Arrington C. M. Carr T. H. Mayer A. R. Rao S. M. (2000). Neural mechanisms of visual attention: object-based selection of a region in space. *J. Cogn. Neurosci.* 2, 106-117. 10.1162/089892900563975 11506651

[B4] Brass M. Derrfuss J. Forstmann B. von Cramon D. Y. (2005). The role of the inferior frontal junction area in cognitive control. *Trends Cogn. Sci.* 9 314–316. 10.1016/j.tics.2005.05.001 15927520

[B5] Calhoun V. D. Pekar J. J. McGinty V. B. Adali T. Watson T. D. Pearlson G. D. (2002). Different activation dynamics in multiple neural systems during simulated driving. *Hum. Brain. Mapp.* 16, 158–167. 10.1002/hbm.10032 12112769 PMC6872105

[B6] Cavanna A. E. Trimble M. R. (2006). The precuneus: a review of its functional anatomy and behavioural correlates. *Brain* 129 564–583. 10.1093/brain/awl004 16399806

[B7] Chikazoe J. (2010). Localizing performance of go/no-go tasks to prefrontal cortical subregions. *Curr. Opin. Psychiatry* 23 267–272. 10.1097/yco.0b013e3283387a9f 20308899

[B8] Derrfuss J. Brass M. Neumann J. von Cramon D. Y. (2005). Involvement of the inferior frontal junction in cognitive control: meta-analyses of switching and Stroop studies. *Hum. Brain Mapp.* 25 22–34. 10.1002/hbm.20127 15846824 PMC6871679

[B9] Derrfuss J. Brass M. von Cramon D. Y. Lohmann G. Amunts K. (2009). Neural activations at the junction of the inferior frontal sulcus and the inferior precentral sulcus: interindividual variability, reliability, and association with sulcal morphology. *Hum. Brain Mapp.* 30 299–311. 10.1002/hbm.20501 18072280 PMC6870901

[B10] Diamond A. (2013). Executive functions. *Annu. Rev. Psychol.* 64 135–168. 23020641 10.1146/annurev-psych-113011-143750 PMC4084861

[B11] Friederici A. D. Rüschemeyer S. A. Hahne A. Fiebach C. J. (2003). The role of left inferior frontal and superior temporal cortex in sentence comprehension: localizing syntactic and semantic processes. *Cereb. Cortex* 13 170–177. 10.1093/cercor/13.2.170 12507948

[B12] Friston K. J. Buchel C. (2000). “Attentional modulation of effective connectivity from V2 to V5/MT in humans,” in *Proceedings of the National Academy of Sciences*, 97, 7591–7596. 10.1073/pnas.97.13.7591 PMC16590 10861020

[B13] Friston K. J. Harrisona L. Pennya W. (2003). Dynamic causal modelling. *NeuroImage* 19 1273–1302. 10.1016/s1053-8119(03)00202-7 12948688

[B14] Goel V. Grafman J. Sadato N. Hallett M. (1995). Modeling other minds. *Neuroreport* 6 1741–1746. 8541472 10.1097/00001756-199509000-00009

[B15] Graydon F. X. Young R. A. Tdss M. D. Genik R. J. Posse S. Hsieh L. (2004). Visual event detection during simulated driving: identifying the neural correlates with functional neuroimaging. 7, 271–286. 10.1016/j.trf.2004.09.006

[B16] Grindrod C. M. Bilenko N. Y. Myers E. B. Blumstein S. E. (2008). The role of the left inferior frontal gyrus in implicit semantic competition and selection: an event-related fMRI study. *Brain Res.* 1229 167–178. 10.1016/j.brainres.2008.07.017 18656462 PMC2566953

[B17] Hadjidimitrakis K. Bakola S. Wong Y. T. Hagan M. A. (2019). Mixed spatial and movement representations in the primate posterior parietal cortex. *Front. Neural Circ.* 13:15. 10.3389/fncir.2019.00015 30914925 PMC6421332

[B18] Hadjidimitrakis K. Breveglieri R. Bosco A. Fattori P. (2012). Three-dimensional eye position signals shape both peripersonal space and arm movement activity in the medial posterior parietal cortex. *Front. Integr. Neurosci.* 6:37. 10.3389/fnint.2012.00037 22754511 PMC3385520

[B19] Howard M. A. Volkov I. O. Mirsky R. Garell P. C. Noh M. D. Granner M. (2000). Auditory cortex on the human posterior superior temporal gyrus. *J. Comp. Neurol.* 416 79–92. 10.1002/(sici)1096-9861(20000103)416:1<79::aid-cne6>3.0.co;2-2 10578103

[B20] Ilieva I. P. Hook C. J. Farah M. J. (2015). Prescription stimulants’ effects on healthy inhibitory control, working memory, and episodic memory: a meta-analysis. *J. Cogn. Neurosci.* 27 1–21. 25591060 10.1162/jocn_a_00776

[B21] Jeong B. Wible C. G. Hashimoto R. Kubicki M. (2009). Functional and anatomical connectivity abnormalities in left inferior frontal gyrus in schizophrenia. *Hum. Brain Mapp.* 30 4138–4151. 10.1002/hbm.20835 19569073 PMC2787802

[B22] Kaas J. H. Stepniewska I. (2016). Evolution of posterior parietal cortex and parietal-frontal networks for specific actions in primates. *J. Comp. Neurol.* 524 595–608. 10.1002/cne.23838 26101180 PMC4689678

[B23] Kim C. Cilles S. E. Johnson N. F. Gold B. T. (2012). Domain general and domain preferential brain regions associated with different types of task switching: a meta-analysis. *Hum. Brain Mapp.* 33 130–142. 10.1002/hbm.21199 21391260 PMC3421461

[B24] Kim H. S. Mun K. R. Choi M. H. Chung S. C. (2020). Development of an fMRI-compatible driving simulator with simultaneous measurement of physiological and kinematic signals: the multi-biosignal measurement system for driving (MMSD). *Technol. Health Care* 28 S335–S345. 10.3233/THC-209034 PMC7369088 32364166

[B25] Kolb B. Whishaw I. Q. (2014). *An Introduction to Brain and Behavior Fourth edition.* New York, NY: Worth, 282–312.

[B26] Levy B. J. Wagner A. D. (2011). Cognitive control and right ventrolateral prefrontal cortex: reflexive reorienting, motor inhibition, and action updating. *Ann. N. Y. Acad. Sci.* 1224 40–62. 10.1111/j.1749-6632.2011.05958.x 21486295 PMC3079823

[B27] Lundstrom B. N. Ingvar M. Petersson K. M. (2005). The role of precuneus and left inferior frontal cortex during source memory episodic retrieval. *NeuroImage* 27 824–834. 10.1016/j.neuroimage.2005.05.008 15982902

[B28] Ma L. Steinberg J. L. Cunningham K. A. Lane S. D. Bjork J. M. Neelakantan H. (2015). Inhibitory behavioral control: a stochastic dynamic causal modeling study comparing cocaine dependent subjects and controls. *NeuroImage Clin.* 7 837–847. 10.1016/j.nicl.2015.03.015 26082893 PMC4459041

[B29] Macaluso E. Frith C. D. Driver J. (2000). Modulation of human visual cortex by crossmodal spatial attention. *Science* 289 1206–1208. 10.1126/science.289.5482.1206 10947990

[B30] Mechelli A. Humphreys G. W. Mayall K. Olson A. Price C. J. (2000). Differential effects of word length and visual contrast in the fusiform and lingual gyri during reading. *Proc. R. Soc. B* 267 1909–1913. 10.1098/rspb.2000.1229 11052544 PMC1690747

[B31] Michon J. A. (1984). “Traffic and mobility,” in *Handbook of Work and Organizational Psychology*, Vol. 2 eds Drenth P. J. D. Thierry H. Willems P. J. de Wolff J. (New York: Wiley), 1165–1196.

[B32] Milner A. D. Goodale M. A. (1998). *The Visual Brain in Action.* Oxford: Oxford University Press.

[B33] Oldfield R. C. (1971). The assessment and analysis of handedness: the Edinburgh inventory. *Neuropsychologia* 9 97–113. 10.1016/0028-3932(71)90067-4 5146491

[B34] Purves D. Cabeza R. Huettel S. A. Labar K. S. Platt M. L. Woldorff M. G. (2008). *Principles of Cognitive Neuroscience.* Sunderland, MA: Sinauer Associates.

[B35] Ruby P. Decety J. (2003). What you believe versus what you think they believe: a neuroimaging study of conceptual perspective-taking. *Eur. J. Neurosci.* 17 2475–2480. 10.1046/j.1460-9568.2003.02673.x 12814380

[B36] Shen H. Li Z. Qin J. Liu Q. Wang L. Zeng L. L. (2016). Changes in functional connectivity dynamics associated with vigilance network in taxi drivers. *NeuroImage* 124 367–378. 10.1016/j.neuroimage.2015.09.010 26363345

[B37] Sundermann B. Pfleiderer B. (2012). Functional connectivity profile of the human inferior frontal junction: involvement in a cognitive control network. *BMC Neurosci.* 13:119. 10.1186/1471-2202-13-119 23033990 PMC3582543

[B38] Tomasi D. Ernst T. Caparelli E. C. Chang L. (2004). Practice-induced changes of brain function during visual attention: a parametric fMRI study at 4 Tesla. *Neuroimage* 23, 1414–1421. 10.1016/j.neuroimage.2004.07.065 15589105

[B39] Wang L. Liu Q. Shen H. Li H. Hu D. (2015). Large-scale functional brain network changes in taxi drivers: evidence from resting-state fMRI. *Hum. Brain Mapp.* 36 862–871. 10.1002/hbm.22670 25338709 PMC6869710

[B40] Yttri E. A. Wang C. Liu Y. Snyder L. H. (2014). The parietal reach region is limb specific and not involved in eye-hand coordination. *J. Neurophysiol.* 111 520–532. 10.1152/jn.00058.2013 24198328 PMC3921407

